# A Social Norms-Based Intervention Improves Dietary Diversity among Women in Rural India: The Reduction in Anemia through Normative Innovations (RANI) Project

**DOI:** 10.3390/nu13082822

**Published:** 2021-08-17

**Authors:** Sameera A. Talegawkar, Yichen Jin, Erica Sedlander, Rohini Ganjoo, Satyaranjan Behera, Loretta DiPietro, Rajiv Rimal

**Affiliations:** 1 Department of Exercise and Nutrition Sciences, Milken Institute School of Public Health, The George Washington University, Washington, DC 20052, USA; yjin@gwu.edu (Y.J.); ldp1@gwu.edu (L.D.); 2 Department of Prevention and Community Health, Milken Institute School of Public Health, The George Washington University, Washington, DC 20052, USA; esedlander@gwmail.gwu.edu (E.S.); rimal@jhu.edu (R.R.); 3 Department of Biomedical Laboratory Sciences, School of Medicine and Health Sciences, The George Washington University, Ashburn, VA 20147, USA; rganjoo@gwu.edu; 4 DCOR Consulting, Bhubaneshwar 751007, India; satyaranjan.dcor@gmail.com; 5 Department of Health Behavior and Society, Bloomberg School of Public Health, Johns Hopkins University, Baltimore, MD 21205, USA

**Keywords:** diet diversity, women of reproductive age, social norms, intervention, anemia

## Abstract

Diet diversity has an important role in the prevention and treatment of anemia. Based on formative research in the community and the theory of normative social behavior, we designed an intervention to improve diet diversity among women of reproductive age. Our study, the Reduction in Anemia through Normative Innovations (RANI) Project, investigated the effect of a social norms-based intervention on diet diversity among women of reproductive age. We randomized villages in Odisha, India, into treatment or control arms, with a minimum of one village buffer between them. We assessed exposure to the intervention by frequency of self-reported images seen from the participatory learning modules, videos watched, and number of hemoglobin tests administered. We assessed diet diversity with the Food and Agriculture Organization’s Minimum Dietary Diversity for Women (MDD-W) questionnaire. We used multiple logistic regression to examine the associations between intervention and diet diversity, adjusting for covariates. Compared with baseline, diet diversity score increased in both treatment and control groups. The odds of having a diverse diet was 47% higher in the treatment group. Higher level of exposure to the RANI intervention was associated with a better diet diversity score, indicating that the intervention was effective in improving diet quality.

## 1. Introduction

Anemia, an important public health concern in low- and middle-income countries, is associated with negative health outcomes, such as increased risk of pre-term delivery, maternal death, and reduced physical capacity in adults [1,2,3,4] as well as poor cognitive development in young children [5]. India has one of the highest global prevalences of anemia, and this is more pronounced among women of reproductive age [6]. According to the India’s National Family Health Survey (NFHS) 2015–2016, 53% of women aged 15–49 years were anemic (defined as a blood hemoglobin concentration <12 g/dL) [7]. The corresponding prevalence in Odisha, a state of India, with significant tribal and rural populations, is 61% [8].

The primary cause of anemia in India is inadequate iron intake, as Indian diets often comprise plant-based foods with low iron bio-availability and few animal products [9]. Lack of financial resources to buy iron-rich foods also affects diet diversity, exacerbating anemia prevalence [10]. Other contributory factors include household size, education status, and prevalence of malaria and other infectious diseases [11,12,13]. India has implemented several iron and folic acid supplementation interventions to reduce the prevalence of iron deficiency anemia. Starting in 1970, the National Nutritional Anemia Prophylaxis Program was initiated in India, which provided iron and folic acid supplements mostly to pregnant and lactating women and pre-school children. Later, in the 1990s, the National Nutrition Policy and the National Plan of Action on Nutrition were launched to develop initiatives and interventions for improving food security, accessibility, and diet quality. These initiatives also provided nutrition and health-education coverage for pregnant women and lactating mothers with the goal of reducing the prevalence of anemia [14]. In 2013, the National Iron+ Initiative, developed by the Adolescent Division of the Ministry of Health and Family Welfare, provided supplementation of iron and folic acid and information about increasing dietary iron intake and awareness of iron-rich foods for children, adolescents, and all women of reproductive age with the goal of decreasing anemia prevalence by 18% in 2020 for women of reproductive age [15]. However, despite these interventions, there has been a negligible shift in the prevalence of anemia. In fact, according to data from NFHS, anemia prevalence has decreased by only 2% among women of reproductive age from 2005 to 2015. In 1998, the prevalence of anemia was 52% among ever-married women aged 15–49 years, similar to the prevalence in 2015 [7,14]. Specifically, hemoglobin levels improved from 2006 to 2016 by 4.5 and 3.2 g/L, and anemia prevalence decreased by 11% and 7.6% in pre-school children and pregnant women, respectively; however, the improvement was minimal in non-pregnant women of reproductive age [16].

The national initiatives implemented to reduce anemia in India have broadly focused on the supply chain—ensuring access to iron supplements and promoting a diverse diet. However, demand-side interventions to change behaviors and social norms surrounding diet and iron consumption are lacking. The Reduction in Anemia through Normative Innovations (RANI) Project [17] is a field trial being conducted in Odisha, India, designed to address this issue. The RANI Project is based on the premise that social norms—which are codes of conduct enacted by a group [18] and sustained through incentives and sanctions [19]—drive behaviors and that to improve healthy behaviors, norms themselves need to be changed. Studies have shown that social norms are associated with intentions to take iron supplements and iron supplement use [20,21]. Prior research on social norms and dietary behaviors has shown that in Australia, social norms assessed by perception of how other people behave regarding eating fast foods or healthy foods affects the actual intakes of these foods [22], but to our knowledge, no studies have quantitatively assessed this relationship in India or elsewhere in Southern Asia. In our qualitative formative research in Odisha, we found that women are the last to eat during mealtimes and therefore often consume meals that are nutrient poor [23]. Further, many women believe that iron supplementation is linked with a “big baby” that results in surgical procedures for delivery, which further deters them from consuming iron supplements during pregnancy [21]. Our formative assessments have also found that decisions regarding women’s health and health-seeking behaviors tend to be imposed by their husbands or in-laws, with the consequence that they may not be appropriately and promptly treated for anemia [23]. For example, women reported that men spent household income on alcohol, which may limit available resources to purchase iron-rich foods. Other barriers include lack of awareness around the high prevalence of anemia in their communities, perceptions that mostly pregnant women have anemia, inadequate knowledge about which foods are rich in iron, and limited awareness about the link between consuming a diverse diet and anemia.

The RANI Project was designed to reduce anemia in all women of reproductive age (15–49 years) by taking a social norms-based approach to increase iron and folic acid supplementation and nutritious foods intake. In this study, we examined over a six-month period the effect of a social norms-based intervention on diet diversity among non-pregnant women of reproductive age. We also hypothesized that higher exposure to the intervention would result in a higher diet diversity score compared with the usual care control group. 

## 2. Materials and Methods

### 2.1. Participants

For this analysis, we used both baseline and a 6-month follow-up (hereby called midline) data from the RANI project (Supplement Figure S1); the detailed description of the RANI project can be found elsewhere [17]. Briefly, villages in Angul, Odisha, were randomly clustered into treatment or control arms, and homes in each cluster were randomly selected using a number generator from a household listing exercise conducted by data collectors in 2019. In each selected home, one woman of reproductive age (15–49 years) was randomly chosen and recruited. Baseline data on sociodemographic information, use of iron and folic acid tablets status, and anemia-related information were collected through one-on-one interviews. At midline, all women received the dietary questionnaire. Of a total of 4110 women in the study at baseline, 157 women were lost to follow-up at midline, 153 women who were pregnant at midline (because diets can change significantly during pregnancy) and 3 women who were missing diet-component data were excluded, resulting in 3797 women as the final analytical sample. For analyses that included both the baseline and midline diet data, we had a total sample size of 918 non-pregnant women with valid diet data, which is smaller than the overall sample because of the planned missingness design at baseline, and it includes women who were at both baseline and midline. 

Informed consent using the local language was obtained by local data collectors, and written permission of legal guardians was also obtained for women under 18 years of age. The study received institutional review board permissions from both Sigma Science and Research in India and the George Washington University in the United States. The study was also reviewed and approved by Indian Council for Medical Research’s Health Ministry’s Screening Committee.

### 2.2. Intervention

The RANI Project uses a social norms-based approach informed by the theory of normative social behavior (TNSB) to increase iron-folic acid supplement use and improve diet diversity [24]. The intervention includes the following three components: (1) participatory education modules delivered through in-person activities and games, (2) locally made videos depicting women overcoming barriers to taking iron supplements and eating an iron-rich diet with the support of their husbands and mothers-in-law, and (3) monthly community-based testing of hemoglobin levels of 15 women in each village, followed by a discussion about trends in anemia and village-level comparisons (based on the hemoglobin readings) with neighboring communities. An example of an interactive activity that helped to link fatigue/physical strength and anemia is a tug-o’-war game among women in the villages. Modules focused on the high prevalence of anemia in the area, symptoms and signs of anemia, iron-folic acid supplementation, and diet quality. Social norms messaging was embedded throughout each module, including raising awareness around unequal gender norms, such as eating last and eating less (Supplementary Table S1). We used both formative research and baseline data to examine existing social norms and to inform social norms messaging [25]. The intervention and the formative research used to design the intervention is described in full detail elsewhere [17,26].

### 2.3. Assessments

Exposure to the intervention at midline was measured by querying women about how often they had, “seen what is shown in the picture”, number of the health communication videos they had seen, and number of hemoglobin tests they had done. Women were shown visual images that were a part of each of the five different participatory education modules (Supplementary Table S1). These images were related to nutritious food intake, symptoms of anemia, effects of anemia, approaches to address anemia, and iron-folic acid supplements to prevent anemia. Each image represented one of five learning modules, which were titled: (i) women in the area, their lifestyle, and their vulnerabilities; (ii) what is anemia, its causes, and symptoms; (iii) effects of anemia; (iv) addressing anemia using various approaches; and (v) supplements for anemia, respectively. Potential responses could include “not seen” (scored 0), “once or twice” (scored 1), or “more than twice” (scored 2). We created a summary overall exposure score by summing up a participant’s responses to all the questions regarding the level of exposure to the five modules. Scores could thus range from 0 to 10, with higher scores indicating greater exposure to the intervention. Women were also asked whether they participated in any games that were a part of the learning modules, and could respond “yes” or “no”; those who responded “don’t know” were re-classified as “no”. To assess the number of health communication videos they had seen, women were shown four videos and queried if they saw each of the videos in the past six months. Affirmative response to each video received a score of 1, which was added up, so the overall score ranged from 0 to 4, with a higher score indicating greater exposure to the intervention. For the number of hemoglobin tests, women were asked how many times they had been tested for anemia as part of the RANI intervention in the past six months, and the responses included never (scored 0), once (score 1), twice (scored 2), three times (scored 3), >3 times (scored 4).

### 2.4. Dietary Assessment

We used the Food and Agriculture Organization’s Minimum Dietary Diversity for Women (MDD-W) questionnaire to assess diet quality [27]. Using the list-based method, local trained interviewers collected intake information for 22 food groups during the previous 24 h. This information was then used to calculate the MDD-W score. The food groups included (i) grains/white roots/tubers/plantains; (ii) pulses (beans, peas, and lentils); (iii) nuts and seeds; (iv) dairy; (v) meat/poultry/fish; (vi) eggs; (vii) dark green leafy vegetables; (viii) other vitamin A-rich fruits and vegetables; (ix) other vegetables; and (x) other fruits. Each woman received 1 point if she reported consumption of foods from each food group, or 0 point if not. We calculated the overall MDD-W score by summing up points for each food group. The possible MDD-W score ranged 0–10, and as recommended, a diverse diet was defined as a score of ≥5 [27].

### 2.5. Covariates

We chose covariates that are associated with anemia, including age, education, caste/tribe status, marital status, mobile phone ownership (a proxy for socio-economic status), body mass index (BMI), currently breastfeeding status (yes vs. no), number of children, iron and folic acid tablet use, and anemia status at baseline in the analyses. Age was analyzed on a continuous scale. Education level was classified as none, primary school, secondary school, higher secondary school, and tertiary school. Caste/tribe status was classified as scheduled caste, scheduled tribe, other backward class (OBC), and none of them. These classifications were based on designations put forth by the Constitution of India [28]. Marital status was categorized as single, married, separated/divorced, and widowed. As a socioeconomic status indicator, mobile phone ownership (yes vs. no) was included in the analysis. Weight and height were measured with standard methods, and BMI was then calculated as weight (in kilograms) divided by square of height (in meters). Iron and folic acid tablet use was categorized as currently taking, taken in the past, and never taken. Anemia status for non-pregnant women was categorized based on hemoglobin levels, assessed by a HemoCue photometer, as normal (hemoglobin ≥ 12 g/dL) or anemic (hemoglobin < 12 g/dL) [29]. Once the participants underwent the hemoglobin tests, they were informed about their hemoglobin count and status by the enumerators. The women who were identified to have anemia were advised to consume iron-rich and nutrient-dense foods, like dark green leafy vegetables, meat, fish, and lentils, on regular basis. The enumerators gave referral slips to women whose hemoglobin levels were below 8 g/dl and advised them to visit a nearby health center for treatment.

### 2.6. Statistical Analysis

Sociodemographic characteristics were reported as mean (standard deviation) or percentage. To compare differences in baseline sociodemographic characteristics between treatment and control groups, *t*-tests and chi-square tests were used for continuous and categorical variables, respectively. Variables with statistically significant differences between treatment and control groups were used as covariates and adjusted for in the regression modeling. Generalized estimating equation (GEE) was used to compare diet diversity scores between baseline and midline stratified by treatment groups, adjusting for age at baseline among a subset sample of non-pregnant women with available diet diversity score information at baseline. The change of diet diversity score from baseline to midline between treatment groups was also examined using difference-in-differences estimate by adding an interaction term for intervention and follow-up time in the GEE model. We tested intervention and exposure to the intervention learning modules and its association with diet diversity score at midline using multiple logistic regression, adjusting for baseline covariates that were significantly different between treatment and control groups. Given the strong association between education and diet in previous literature [30,31], we also performed a sensitivity analysis by including education level in the model. All analyses were performed with SAS 9.4, and a two-tailed statistical significance level of 0.05 was used.

## 3. Results

At baseline, the mean age of the study population was 31 years (SD = 8.8), with mean BMI 21.4 kg/m^2^ (SD = 3.7). The majority of women were married (80%), had low levels of education (less than 10 years of formal schooling), and about 40% of women belonged to the scheduled caste/tribe. While two-thirds of women had anemia (according to their HemoCue reading), only 6% of women reported taking iron and folic acid supplements, and 43% of women had a diet diversity score ≥5. Between the treatment and control groups, women in the treatment group tended to be older, had a higher BMI, and were less likely to be from the scheduled tribe (*p* for all <0.05). There was no significant difference at baseline between treatment and control groups for diet diversity and anemia status (Table 1).

Compared with baseline, women in the control group had an 8% increase at midline in MDD-W score (*p* < 0.01), and women in treatment group had 13% increase (*p* < 0.001) (Table 2). The relative increase in the diet diversity score in the treatment arm was not significantly different from that in the control arm. Among all MDD-W components, in both treatment and control groups, significant increases from baseline to midline were observed in the consumption of pulses, nuts and seeds, dairy, meat and poultry or fish, eggs, and dark green leafy vegetables. We also saw increases in consumption of vegetables and fruits. However, we did not see significant improvements from baseline to midline in vitamin A-rich foods.

At baseline, because age, tribe status, and BMI in the control group were different from those in the treatment group, we adjusted for these three variables in the regression model examining associations between intervention and diet diversity at midline (Table 3). Women in the treatment group had 47% higher odds of having MDD-W score ≥5 than women in control group (odds ratio (OR) = 1.47, 95% confidence interval (CI): 1.29–1.68, *p* < 0.001). Approximately 88% of women in the treatment arm reported some exposure to at least one intervention module in comparison to 12% in the control group. Women who had greater exposure to the intervention modules had higher odds of having MDD-W score ≥ 5 (OR = 1.10, 95% CI: 1.07–1.13, *p* < 0.001). Specifically, women who reported seeing the “food intake” pictures more than twice had two times the odds of having MDD-W score ≥5 (more diverse diet) than women who never viewed these pictures (OR = 2.09, 95% CI: 1.64–2.67, *p* < 0.001), and viewing the pictures on “iron-folic acid (IFA) supplements for anemia” more than twice was associated with 2.3 times higher odds of having a more diverse diet compared to not viewing the pictures (OR = 2.28, 95% CI: 1.67–3.12, *p* < 0.001). Higher exposure to other non-diet or IFA supplement-related pictures and participating games related to learning modules were also positively associated with diet diversity. Moreover, exposure to the health communication videos was positively associated with diet diversity (OR = 1.15, 95% CI: 1.10–1.20, *p* < 0.001). Lastly, more frequent anemia testing was associated with a more diverse diet. All associations remained statistically significant after additional adjusting for education.

## 4. Discussion

Our study investigated whether a social norms-based intervention was associated with increased diet diversity. Our results indicated that among non-pregnant women of reproductive age living in rural Odisha, India, a social norms-based intervention was associated with a more diverse diet in the treatment group compared to the control group. Our dose-response analysis demonstrated that higher exposure to the intervention modules was also associated with better diet diversity. We should note, however, that a difference-in-difference test showed that improvements in diet diversity were not significantly different in the treatment arm as compared to the control arm.

In our intervention, diet quality improved significantly at midline as compared to baseline in both control and treatment groups. An important reason for this could be availability of foods across different seasons. One meta-analysis and systematic review reported that seasonality played an important role in the intake of various food groups, noting seasonal differences in consumption of vegetables, fruits, eggs, and meat as well as energy intake [32]. In our study, we conducted the baseline survey in the summer-monsoon months of June through September and the midline assessments in winter-spring months of January through March. Thus, our findings likely reflect the different types of foods consumed between baseline and midline.

In the Pune Maternal Nutrition Study, pregnant Indian women reported dietary variations in different seasons, with a higher percent of women meeting dietary recommendations in the winter compared to summer or monsoon seasons [33]. Similar findings have been reported in rural Pakistani households, where diverse foods are produced in different seasons. Even with family income being relatively steady throughout the year, compared to summer, the food variety score, diet diversity score, and energy intake were higher in winter [34]. Food prices and the fluctuation in household incomes across the year also play a role in food accessibility and food selection [35]. In addition, dietary tradition or norms dictate that different foods are consumed across seasons; for example, more high-calorie foods are commonly consumed in winter than other seasons [34,36].

Our study indicated a “dose response” related to various intervention components, which further highlights the effectiveness of a social norms-based intervention in improving diet quality. The theory of normative social behavior posits that behaviors can be changed by social norms, which refer to people’s beliefs about what others do (also called descriptive norms) and their perceptions about what is expected of them (injunctive norms) [24]. When women believe that others in their community are adopting these behaviors (descriptive norms) and that others expect them to adopt these behaviors (injunctive norms), women may be more likely to adhere to them (e.g., stick to a diverse diet). 

Our midline analysis shows that a social norms-based intervention can increase diet diversity to prevent anemia. Previous literature has demonstrated the critical role of nutrition education intervention programs on improving diet quality and hemoglobin level. In a study conducted in three rural villages in Maharashtra, India [32], a field intervention comprising activities such as informal meetings, demonstration of recipes with green leafy vegetables, and kitchen garden activities reported that higher participation in these activities was associated with a higher frequency of consumption of green leafy vegetables and higher hemoglobin results. Another nutrition education intervention conducted among women in a rural hill area in Uttarakhand State, India, showed significant improvements in nutritional knowledge in the intervention group [37].

It is important to note that our social norms-based intervention targeted not only women of reproductive age but also mothers-in-law and husbands. Many learning modules were viewed by women (the focal audience) and by their husbands and mothers-in-law. This perhaps emphasizes the social component of social norms: behavior change is more likely to occur among the target audience when her social network members are also targeted for change. In rural India, husbands and in-laws play a large role in key household decisions, and women themselves tend not to prioritize their own health and well-being, as was also demonstrated in our own formative research [23]. Although women take care of the whole family, only a small fraction of family resources were accessible to them for their own health. Therefore, it is essential to include husbands and mothers-in-law in the intervention program to ensure that women adopt and have high adherence to positive behavioral changes. We also embedded social norms messaging throughout learning modules, with statements like, “many women in your community are starting to eat a diet rich in iron and taking care of themselves, too”.

### Limitations

Our study has some limitations. Diet diversity at baseline was measured only in a small subset of the study population. However, using an intentional missingness design, this subset was selected at random, and therefore we can infer that the findings would be generalizable to the entire study population. To reduce participant time and burden, we did not conduct a 24-h dietary recall measure, which could have provided us a more comprehensive dietary-intake assessment, including nutrient-intake estimates. The diet diversity score was calculated based on the self-report of foods and food groups by the study participants and therefore is subject to measurement error and social desirability bias [38]. However, in our study, any resulting misclassification because of this would apply equally to the control and intervention arms of the study population, resulting in type II error. 

Additionally, we did not collect information on diet-related social norms, and therefore we were unable to examine whether the RANI intervention led to women and their families adopting healthy diet-related social norms. Lastly, because no food or money was provided as part of the intervention, financial constraints may have been a barrier and limited the accessibility to a more diverse diet. Despite this, we still observed significant changes, suggesting that the social norms-based activities were likely the primary source of influence.

Finally, we note that a small portion (about 10%) of those in the control arm also reported some exposure to the intervention. It is difficult to tell how much of this is due to contamination effects and how much of it is because of an acquiescence bias, the tendency to reply in the affirmative when asked if one has seen a particular message, video, or an intervention component. In this cluster-randomized design, we minimized contamination by having a one-village buffer between treatment and control clusters. Residents of one cluster, however, could have attended sessions held in another by traveling to intervention sites. Nevertheless, the fact that we found significant differences between treatment and control arms leads us to believe in the potency of the intervention.

Despite these limitations, our study has some strengths. We used a randomized sampling procedure so that women included in the study were representative of women in the study area. Given its rigorous random sampling design, our results have high internal validity. However, since our study focused on rural communities, findings may not be generalizable to the entire state of Odisha or India. We administered a multi-component intervention that emphasized nutrition and anemia-related education and behavioral change at individual, family, and community levels. Furthermore, we investigated not only treatment/control intervention group but also the exposure levels to the intervention to capture any dose-response effect of frequency of exposure to treatment on diet diversity. 

## 5. Conclusions

Our results showed that in our study population of women of reproductive age, diet diversity improved at midline (six months), as compared to baseline, with marginally higher scores in the treatment group. Women with higher exposure to the intervention reported better diet quality. These results emphasize the initial effectiveness of a social norm-based intervention for improving dietary quality. Subsequent research using end-line data will elucidate how additional intervention exposure (six more months) affects diet diversity.

## Figures and Tables

**Table 1:** Sociodemographic characteristics in control and treatment group at baseline among women of reproductive age in Reductions in Anemia through Normative Innovations (RANI) project, Odisha, India.

	Overall (*n* = 3797)	Control (*n* = 1896)	Treatment (*n* = 1901)	*p*
Age, years	30.7 (8.8)	30.4 (8.7)	31.1 (8.8)	0.027
Body mass index, kg/m^2^, *n* = 3629	21.4 (3.7)	21.2 (3.7)	21.6 (3.7)	0.003
Mobile phone owner	47.6	48.6	46.7	0.224
Education				0.527
None	18.9	18.2	19.5	
Up to class 7	38.0	38.5	37.4	
Class 8–10	31.0	30.5	31.5	
Class 11–12	9.1	9.8	8.5	
More than class 12	3.1	3	3.2	
Marital status				0.776
Single	15.6	15.9	15.2	
Married	80.2	79.7	80.6	
Separated/divorced	0.8	0.7	0.9	
Widowed	3.5	3.6	3.3	
Scheduled caste, tribe, and other backward class status, *n* = 3790				<0.001
Scheduled Caste	13.6	11.4	15.8	
Scheduled Tribe	28	31.6	24.4	
Other Backward Class	56.3	55.1	57.4	
None of them	2.1	1.9	2.4	
Iron and folic acid tablet use				0.217
Never taken	21	20	22	
Currently taking it	5.8	6.2	5.4	
Took in the past but not currently	73.2	73.8	72.6	
Current breastfeeding, *n* = 3795	21.2	21.5	20.9	0.690
Number of children	1.7 (1.2)	1.7 (1.3)	1.7 (1.2)	0.562
MDD-W score ≥ 5, *n* = 952	43.1	42.1	44	0.562
Anemia (hemoglobin < 12 g/dL), *n* = 3783	64.2	65.4	63.0	0.123

**Table 2:** Comparison of diet diversity between baseline and midline among non-pregnant women of reproductive age in Reductions in Anemia through Normative Innovations (RANI) Project, Odisha, India.

	Control, *n* = 456 ^a^			Treatment, *n* = 462 ^a^			*p* for d-in-d ^b^
	Baseline, %	Midline, %	*p*	Baseline, %	Midline, %	*p*	
Diet diversity score ≥5	42.8	51.1	0.004	44.4	57.4	<0.001	0.274
*MDD-W components*							
Grains/white roots/tubers/plantains	99.8	99.8	1	99.8	99.8	1	1
Pulses	78.3	83.8	0.023	76.6	86.4	<0.001	0.227
Nuts and seeds	20.8	27.6	0.010	24.7	33.6	<0.001	0.552
Dairy	24.3	16.7	0.003	25.8	18.0	<0.001	0.975
Meat/poultry/fish	20.8	28.3	0.008	16.5	28.4	<0.001	0.234
Eggs	7.2	9.0	0.531	6.7	12.6	<0.001	0.031
Dark green leafy vegetable	37.5	44.1	0.036	38.7	45.2	0.044	0.980
Vitamin A-rich vegetables and fruits	37.9	32.2	0.070	39.0	38.7	0.952	0.223
Other vegetables	86.8	93.2	0.001	90.5	95.2	0.003	0.552
Other fruits	16.9	22.2	0.026	17.5	24.7	0.006	0.636

Note: Model adjusted for baseline age. ^a^ Smaller sample size (*n* = 918) in this analysis includes both baseline and midline diet data because of planned missingness design at baseline. ^b^ Difference-in-differences (d-in-d) estimate test for changing of diet diversity score from baseline to midline comparing treatment to control group.

**Table 3:** Associations between intervention and exposure to intervention learning modules with high diet diversity (Minimum Dietary Diversity for Women (MDD-W) ≥5) at midline among non-pregnant women of reproductive age in Reductions in Anemia through Normative Innovations (RANI) project, Odisha, India.

	OR	95% CI	*p*
Treatment vs. control	1.47	1.29, 1.68	<0.001
Overall exposure to learning modules ^a^	1.10	1.07, 1.13	<0.001
Visual images of learning modules			
Food intake			
Not seen	Reference		
Once or twice	1.34	1.16, 1.55	<0.001
More than twice	2.09	1.64, 2.67	<0.001
Symptoms of anemia			
Not seen	Reference		
Once or twice	1.53	1.32, 1.78	<0.001
More than twice	1.99	1.54, 2.58	<0.001
Effects of anemia			
Not seen	Reference		
Once or twice	1.42	1.22, 1.65	<0.001
More than twice	1.96	1.51, 2.54	<0.001
Approaches to address anemia			
Not seen	Reference		
Once or twice	1.56	1.34, 1.82	<0.001
More than twice	1.93	1.33, 2.81	<0.001
Iron-folic acid supplements for anemia			
Not seen	Reference		
Once or twice	1.48	1.27, 1.73	<0.001
More than twice	2.28	1.67, 3.12	<0.001
Participation in learning module games	1.40	1.19, 1.65	<0.001
Number of hemoglobin tests as part of the RANI intervention			
Never	Reference		
Once	1.35	1.16, 1.58	<0.001
Twice	1.36	1.08, 1.72	0.008
Three times	1.60	1.05, 2.43	0.030
Greater than 3 times	2.36	1.50, 3.73	<0.001
Exposure to health communication videos	1.15	1.10, 1.20	<0.001

Note: Multiple logistic regression models were used adjusting for age, tribal status, and body mass index at baseline. ^a^ Intervention learning modules included 10-h interactive modules on anemia and nutrition-related knowledge.

## Data Availability

The dataset for this manuscript has been made available in the George Washington University repository, GW ScholarSpace (https://scholarspace.library.gwu.edu/work/gx41mj67p) (accessed on 1 August 2020).

## Supplementary Materials

The following are available online at https://www.mdpi.com/article/10.3390/nu13082822/s1: Supplementary Figure S1, Participants recruitment flow chart; Supplementary Table S1, Learning modules contents and embedded social norms messaging and visual images of learning modules for assessing exposure to intervention.
