# Supplementary material for: A Social Norms-Based Intervention Improves Dietary Diversity among Women in Rural India: The Reduction in Anemia through Normative Innovations (RANI) Project
Source: Nutrients. 2021 Aug 17;13(8):2822. doi: 10.3390/nu13082822 (PMC8401778; doi:10.3390/nu13082822)
Supplement: Supplementary file 1 [file nutrients-13-02822-s001.zip › nutrients-1277299-SI.pdf]

Supplement Figure S1. Participants recruitment flow chart

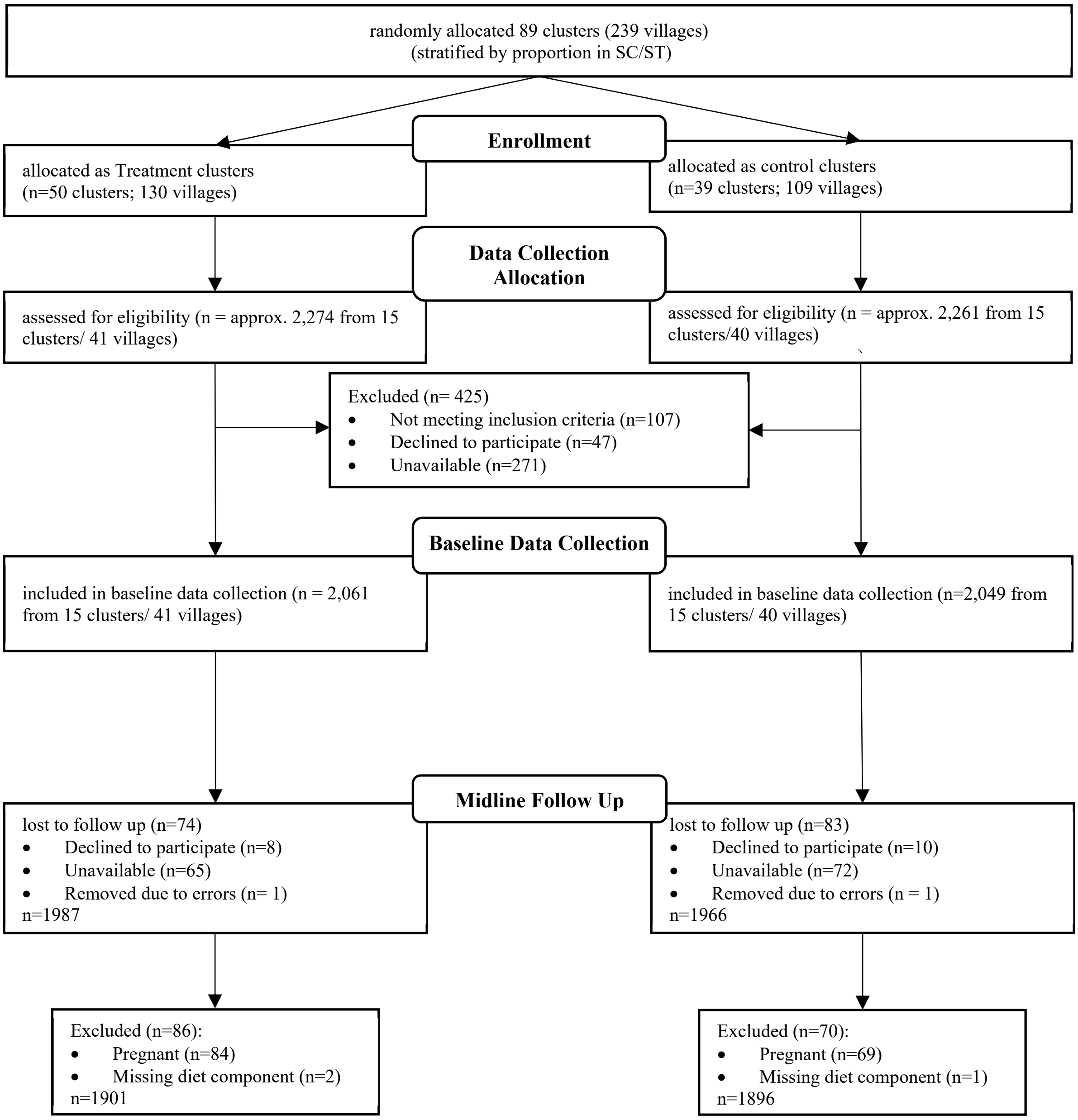

Supplementary Table S1. Learning modules contents and embedded social norms messaging and visual images of learning modules for assessing exposure to intervention.

Learning modules	Content	Social norms messaging	Visual images
Food intake	Both men and women require a good nutritious diet since both do a lot of work on a daily basis. Eating nutritious food will ensure that they prevent serious deficiencies like malnutrition and anemia	Many women are now ensuring that they take their meals on time and keep aside a portion of nutritious food for themselves/ Many men have started ensuring that they bring adequate iron rich food to the house. /Many mother-in-laws have started helping their daughter in laws in household work and also ensuring that they get adequate rest and a nutritious diet.	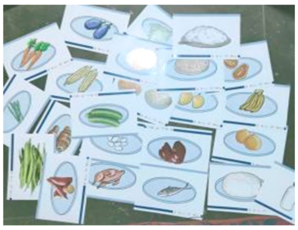
Symptoms of anemia	Women often take symptoms of anemia like fatigue, dizziness etc. as a routine part of their lifestyle. However, these can be symptoms of anemia and needs to be addressed with immediate testing, IFA supplementation and an iron rich diet.	More and more women in the community are addressing their own health issues by getting themselves tested and seeking health care.	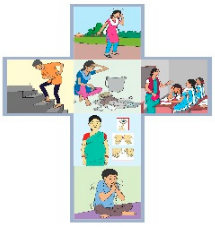
Effects of anemia	If anaemia is not treated, it could lead to serious health issues. An anemic mother could give birth to a low birth weight baby, who could grow into an anaemic adolescent and later become an anaemic mother. So anemia has a long term impact and needs to be addressed immediately.	More and more women in the community are addressing their anemia by getting themselves tested, taking IFA supplements and eating an iron rich diet./More and more mothers and mother in laws are taking care of the health of their daughter/daughter in laws.	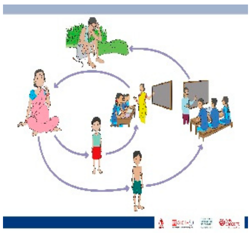

Approaches to address anemia	Often women live their lives to meet goals that are set and guided by others, such as marrying early or having children immediately after marriage. l These “others” can be their family members, elders, neighbours, community members, etc. They can either prove to be enablers or become barriers in our lives. Everyone should have the opportunity to choose their life goals, with their eyes open. One should be able to participate in all important decisions related to their lives.	More and more women in the community are taking decisions about their life/health themselves by attending RANI sessions, getting themselves tested, taking IFA supplements and an iron rich diet	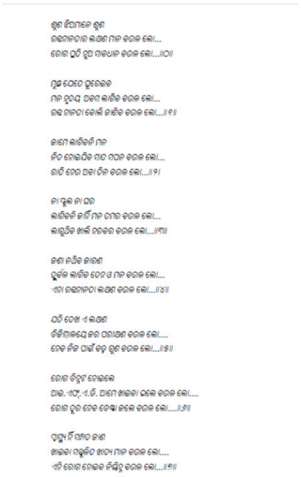
Iron folic acid supplements for anemia	Taking IFA tablets regularly is as important as sunlight, air, water and food for us. IFA tablets help in preventing anaemia.	More and more women in this village, whether they are pregnant or not, are starting to take IFA tablets	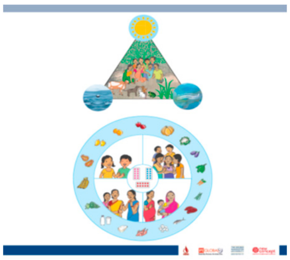
